# Intracranial Angioplasty with Enterprise Stent for Intracranial Atherosclerotic Stenosis: A Single-Center Experience and a Systematic Review

**DOI:** 10.1155/2021/6645500

**Published:** 2021-04-17

**Authors:** Bowen Sun, Chao Xu, Pei Wu, Man Li, Shancai Xu, Chunlei Wang, Xiangyu Liu, Yeping Ling, Huaizhang Shi

**Affiliations:** ^1^Department of Neurosurgery, The First Affiliated Hospital of Harbin Medical University, Harbin, Heilongjiang Province, China; ^2^Department of Neurology, The First Affiliated Hospital of Harbin Medical University, Harbin, Heilongjiang Province, China; ^3^Department of Neurology, Shenzhen Longhua District Central Hospital, Shenzhen, Guangdong Province, China

## Abstract

**Background:**

The high rate of periprocedural complications for the endovascular stent procedure in the Stenting Versus Aggressive Medical Management Therapy for Intracranial Arterial Stenosis (SAMMPRIS) trial resulted in it being less recommended than medical therapy to treat intracranial atherosclerotic stenosis (ICAS). Because Enterprise stent use might reduce the incidence of complications in ICAS treatment compared to other frequently used stents, this paper evaluated the safety and effectiveness of the Enterprise stent for the treatment of ICAS.

**Methods:**

We performed a comprehensive literature search for reports on intracranial angioplasty using the Enterprise stent for ICAS treatment from the earliest date available from each database to May 2020 for PubMed, EMBASE, Web of Science, Cochrane, and Clinical Trials databases. We also reviewed the single-center experience of the First Affiliated Hospital of Harbin Medical University. We extracted information regarding periprocedural complications, procedure-related morbidity, mortality, immediate angiographic outcome, and long-term clinical and angiographic outcomes, among others. Event rates were pooled across studies using random-effects or fixed-effects models depending on the heterogeneity.

**Results:**

Five hundred fifty-seven patients with 588 lesions from seven studies, including the institutional series, were included in the analysis. The incidence of stroke or death within 30 days was 7.4% (95% confidence interval (CI), 5.5%–10.1%). The incidence of ischemic stroke or TIA in the territory of the qualifying artery beyond 30 days and during follow-up was 3.2% (95% CI, 1.1%–9.5%). The incidence of in-stent restenosis was 10.1% (95% CI, 4.6%–22.2%), and the incidence of symptomatic restenosis was 4.1% (95% CI, 1.7%–9.9%).

**Conclusions:**

Intracranial angioplasty utilizing the Enterprise stent for ICAS treatment was relatively safe and effective but required further verification using additional sources for evidence.

## 1. Introduction

For decades, intracranial atherosclerotic stenosis (ICAS) has been a major risk factor for ischemic stroke worldwide, especially in Asian populations [1, 2]. The Chinese Intracranial Atherosclerosis (CICAS) trial reported that, in China, 46.6% of patients with cerebral ischemia symptoms exhibited ICAS [3]. According to current guidelines, the primary treatment for ICAS is medical therapy [4]. However, for ICAS patients with severe stenosis and symptoms of cerebral ischemia (>70%), the stroke recurrence rate after receiving aggressive medical therapy (AMT) exceeds 20% annually [5]. Percutaneous transluminal angioplasty and stenting (PTAS) has been regarded as an effective alternative method to treat severe ICAS [6].

In the past decade, two large randomized controlled trials (RCTs), Stenting Versus Aggressive Medical Management Therapy for Intracranial Arterial Stenosis (SAMMPRIS) [7] and Vitesse Intracranial Stent Study for Ischemic Stroke Therapy (VISSIT) [8], indicated that AMT was the preferred ICAS treatment relative to PTAS due to high rates of periprocedural complications associated with PTAS. In the SAMMPRIS trial, the 30-day incidence of stroke or death in the stent group was 14.7%, which was significantly higher than 5.8% for the AMT group and was partly due to the use of the Wingspan stent [9, 10]. The Wingspan stent is the only stent currently approved by the Food and Drug Administration for the treatment of ICAS [11]. However, concerns have been raised that its rigidity and open-cell design with high radial force could be related to the higher perioperative complication rate observed in previous trials [12].

Meanwhile, other stent varieties not originally designed to treat ICAS have been used for off-label treatment of ICAS. Some of these off-label stents have achieved satisfactory results, including other self-expanding stents such as the Enterprise stent (Codman Neurovascular, Raynham, Massachusetts, USA), balloon-expandable stents, and drug-eluting stents [9, 13–20]. Among these varieties, the Enterprise stent, which has a closed-cell design, special bearing system, and lower radial force, provides an attractive option to treat ICAS [9, 13–17]. This preference is supported by the fact that the Enterprise stent is associated with fewer perioperative complications, and it can reach many lesions that other stents cannot due to the inclusion of microcatheters [9, 13–17]. However, only a few case series with limited sample sizes have been published that assess the treatment of ICAS using the Enterprise stent [9, 13–17]. No systematic review has been conducted. Due to the advantages mentioned above, it is possible that the Enterprise stent might become one of the predominant devices used to treat ICAS. Therefore, it is necessary to further verify the safety and effectiveness of the Enterprise stent in the treatment of ICAS. Thus, we reported on the outcomes of using the Enterprise stent to treat ICAS in a high-volume center and systematically reviewed all relevant literature.

## 2. Materials and Methods

### 2.1. Institutional Series

#### 2.1.1. Patient Population and Lesion Characteristics

We conducted a study of case series from a single center and reported the results according to the Preferred Reporting Of CasE Series in Surgery (PROCESS) guidelines [21]. We retrospectively collected data from consecutive patients who underwent PTAS using the Enterprise Stent to treat ICAS in our institution (First Affiliated Hospital of Harbin Medical University, Harbin, China) from June 13, 2017, to April 3, 2020. The First Affiliated Hospital of Harbin Medical University is located in Harbin, Heilongjiang Province, in northeastern China. The area is located at a high altitude, cold climate, and a high incidence of cerebrovascular diseases. The center sees more than 1,500 cases of cerebrovascular diseases each year, including PTAS, and treats approximately 200 ICAS patients per year.

Written informed consent was obtained from each patient, and all study procedures were approved by the institutional ethics review board (Ethical approval: number ID, 20D0051). The inclusion criteria included the following. (1) The patient received a diagnosis of ICAS with a degree > 70%, which was confirmed by digital subtraction angiography (DSA), using the same methods as the Comparison of Warfarin and Aspirin for Symptomatic Intracranial Arterial Stenosis (WASID) trial [5]. (2) The patient exhibited recurrent transient ischemic attack (TIA) or ischemic stroke despite receiving AMT. (3) Hypoperfusion was present in the region surrounding the qualifying artery that was confirmed by computed tomography perfusion (CTP) and magnetic resonance imaging (MRI). The exclusion criteria included the presence of (1) complete occlusion of the cerebral artery; (2) stenosis caused by nonatherosclerotic factors; (3) ICAS combined with other intracranial diseases such as cerebral hemorrhage, malformation, aneurysm, moyamoya disease, and intracranial tumors; and (4) occurrence of a new ischemic stroke within two weeks before admission, as verified by diffusion-weighted imaging (DWI).

#### 2.1.2. Procedures

All patients received dual antithrombotic therapy for a minimum of five days before stenting, as well as oral aspirin (100 mg/d) and clopidogrel (75 mg/d), and their platelet inhibition rate was assessed. Preoperative blood pressure was maintained 15% lower than the baseline blood pressure. Patients remained under general anesthesia during the stent procedure, and heparin was infused following induction of anesthesia. A 6F catheter was inserted into the common carotid or vertebral artery via the femoral artery using the Seldinger method. After femoral artery puncture, heparin was injected intravenously, and heparin saline was continuously infused during the procedure to ensure that the activated clotting time was between 150 and 250 seconds. Subsequently, angiography was performed to assess stenosis lesions and blood flow compensation.

We calculated the diameter and length of stenosis lesions utilizing both three-dimensional rotation and two-dimensional imaging. Under the guidance of the path map, a Traxcess 14 (Microvention, USA) microguidewire was used with the Excelsior SL-10 (Stryker, Kalamazoo, Michigan, USA) microcatheter to super select the distal end or branch of the blood vessel through the lesion area, and the microguidewire was withdrawn. After confirming the true lumen of the blood vessel, the Transcend 300 microguidewire (Stryker) was inserted to withdraw the microcatheter. Then, the Gateway balloon catheter (Boston Scientific, USA) was sent along the Transcend Floppy 300 guidewire to the distal end of the lesion area, such that the size of the balloon catheter reached 80% of the lesion stenosis and the balloon was expanded slowly. After satisfactory balloon expansion, it was withdrawn, and the Select Plus microcatheter (Stryker) was inserted along the exchange guidewire. The Enterprise stent (Codman) was delivered to the lesion area through the microcatheter. It was necessary that the stent completely cover the lesion, and the length needed to be greater than 3-5 mm at both ends of the lesion area. After stenting, angiography was performed to assess the procedure results and rule out thrombosis in the stent or distal thromboembolism. The patient's blood pressure was maintained at 120-140 mmHg after treatment, and the patient continued to receive dual antithrombotic therapy, including clopidogrel (75 mg/d) for six weeks and aspirin (100 mg/d) for at least six months. Patients received follow-up telephone calls 30 days after the procedure.

#### 2.1.3. Data Collection and Outcomes

We reviewed medical records and extracted basic information concerning the patients and lesions, including demographic data, lesion characteristics, clinical presentations, modified Rankin scale (mRS) scores, and the degree of arterial stenosis. Indicators related to stent placement were recorded, including the size of the balloon catheter and Enterprise stent, complications within 30 days after stenting, and postoperative angiography results.

The safety was assessed by noting the occurrence of adverse events within 30 days after stenting, including TIA, stroke, or death, based on the guidelines of the SAMMPRIS trial. Besides, we recorded the rate of technical success, which was defined as remaining stenosis of 50% or less as measured by immediate postoperative angiography, which indicated the precise release of the Enterprise stent into the lesion area.

### 2.2. Literature Review

#### 2.2.1. Study Protocol and Search Strategy

We conducted a systematic review of relevant literature, following the Preferred Reporting Items for Systematic Reviews and Meta-Analyses (PRISMA) guidelines [22]. The study was registered in the International System of Review Prospective Register (PROSPERO, CRD42020183509). With the assistance of an experienced librarian, we searched PubMed, EMBASE, Web of Science, Cochrane, and Clinical Trials databases. Keywords, including “intracranial arteriosclerosis,” “cerebral arterial diseases,” “cerebral arteries,” “internal carotid,” “vertebrobasilar arteries,” “middle cerebral artery,” “stents,” and “Enterprise” were used in both “AND” and “OR” combinations, as described in Table S1. The literature search period was from the earliest date available for each database to May 2020. All studies reporting ICAS patients treated with the Enterprise stent were selected.

#### 2.2.2. Trial Selection

Studies reporting an ICAS case series treated with Enterprise stents with a sample size greater than five met the initial inclusion criteria. We reviewed all potentially qualified studies with results specifically related to safety or effectiveness. We excluded studies that did not provide perioperative complication rates and technical success rates. Duplicate studies were moved. Non-English articles, conference abstracts without full text, and case series combined with complete occlusion of the cerebral artery and moyamoya disease also were excluded.

#### 2.2.3. Data Extraction

Two investigators (BWS and CX) independently extracted the following information from each eligible study: the total number of patients and lesions treated with the Enterprise stent, pretreated and posttreated mean stenosis degree, technical success rate, intraprocedural complications, frequency of stroke, TIA, or death within 30 days after implantation of the Enterprise stent, frequency and mean duration of clinical and imaging follow-ups, frequency of stroke, TIA or death in the territory of the qualifying artery beyond 30 days, and the in-stent restenosis (ISR) and symptomatic ISR rates. The presence of ISR was indicated by a greater than 50% residual stenosis in the stent after placement as assessed by follow-up DSA or computed tomographic angiography (CTA) examination.

#### 2.2.4. Qualitative Assessment

Two investigators (BWS and CX) independently assessed the quality of the included literature, and a third investigator resolved any disagreements. Literature quality was assessed using a modified version of the Newcastle-Ottawa Quality Assessment Scale (NOS) [23], which was specifically designed to assess the quality of nonrandomized studies, such as case-control studies and cohort studies. We evaluated the quality of each study based on three aspects, including (1) selection of the study groups, (2) comparability of the study groups, and (3) achievement of the outcome of interest. Specific evaluation details are shown in Table S2.

### 2.3. Statistical Analysis

All statistical analyses were performed using R software (version 3.6.1, R Core Team, Vienna, Austria). Standard descriptive statistics were used for the institutional series. Continuous data were presented as means ± standard deviation. Categorical data were presented as percentages. Since all included studies were noncomparative studies, we calculated incidence rates rather than relative risks or mean differences. The cumulative incidence and 95% confidence interval (CI) for all events were recorded, and cumulative outcomes were calculated. Subgroup analyses were conducted based on the anterior circulation (AC) and posterior circulation (PC) of the cerebral arteries. For the pooled analysis, event rates were summarized using a random-effects model if heterogeneity was significant; otherwise, a fixed-effects model was used [24]. Study heterogeneity was evaluated using the *I*^2^ statistic. *I*^2^ values of 0-25%, 26-50%, 51-75%, and>75% indicated light, low, moderate, and high heterogeneity, respectively [25]. If any apparent heterogeneity was observed, a sensitivity analysis was used to explore the source of the heterogeneity. Visualization using a funnel plot was employed to assess publication bias when there were sufficient numbers of eligible studies to create the plot. Asymmetric funnel plots are suggestive of publication bias.

## 3. Results

### 3.1. Institutional Series

#### 3.1.1. Patient Population and Lesion Characteristics

Three hundred twenty-one ICAS patients received PTAS treatment at our center from June 13, 2017, to April 3, 2020. After excluding ineligible participants, 104 patients (mean age, 58.61 ± 9.32 years) with 105 stenosis lesions were incorporated into the present study (60 male patients, 57.69%). The screening flowchart is seen in Figure S1. All relevant patient data are shown in Table 1. Forty-seven (44.76%) lesions were located in the AC (13 in the intracranial segment of the internal carotid artery, ICA (12.38%); and 34 in the middle cerebral artery, MCA (32.38%)). Fifty-eight (55.24%) lesions were located in the PC (15 in the intracranial segment of the vertebral artery, VA (14.28%); and 43 in the basilar artery, BA (40.95%)). Twenty-one (20.19%) patients were admitted to the hospital for TIA and 83 (79.81%) for stroke. The frequency of preoperative mRS scores were as follows: 0, 17 (16.35%); 1, 69 (66.35%); 2, 16 (15.38%); and 3, 1 (0.96%). The preoperative degree of arterial stenosis was 87.13 ± 7.80%, as determined by DSA.

#### 3.1.2. Immediate Angiographic and 30-Day Outcomes

All 105 stents met the technical success criteria, resulting in a 100% success rate, and postoperative stenosis averaged 27.31 ± 8.89% (Table 1). Within 30 days after stent placement, 7 (6.73%) patients developed stroke or died, 4 (3.81%) patients experienced an ischemic stroke, and 3 (2.88%) patients developed a hemorrhagic stroke. One patient with a hemorrhagic stroke died, yielding a total mortality rate of 0.95%. One patient with a right MCA stenosis experienced hyperperfusion cerebral hemorrhage on the second day after stent placement and died, despite immediate symptomatic treatment. One patient with stenosis at the end of the left ICA developed a subarachnoid hemorrhage 12 hours after the stent was implanted. This patient subsequently underwent decompressive craniectomy, gradually achieved full recovery, and exhibited a mRS score of 3 on day 30 after the procedure. One patient with cerebellar hemorrhage did not present any visible symptoms but scored 1 on the mRS on day 30 following the procedure. Three patients with BA stenosis and one patient with MCA stenosis developed a perforating infarction within 30 days after the procedure, but the symptoms were not severe. After receiving antiplatelet therapy, their symptoms improved, and their mRS scores were 0-2 at the 30-day telephone follow-up interview.

### 3.2. Systematical Review

#### 3.2.1. Search Results

**Figure 1:**
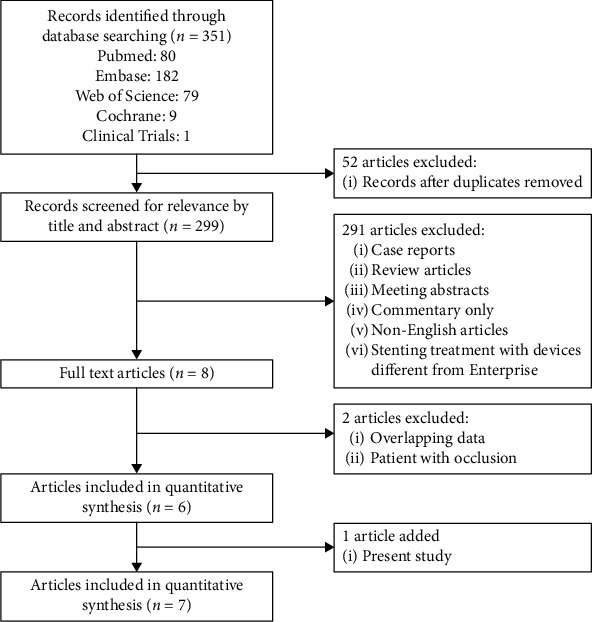
Flowchart shows study selection procedure. 7 studies were included in this systematic review.

The literature selection process is shown in Figure 1. The initial database search identified 351 citations. Fifty-two duplicates were excluded, and 291 articles were excluded after reading the titles and abstracts, leaving eight articles. Two articles presented overlapping data, and we chose to include the article with the longest study duration and the largest number of cases. One additional study was excluded for including only patients with complete occlusion of the cerebral artery. Therefore, seven studies, including the institutional series, were included in the systematic review.

#### 3.2.2. Characteristics of Included Studies

The baseline information for all studies is shown in Table 2. Studies were published between 2012 and 2019. All included studies were retrospective observational case studies that lacked comparisons to other treatments as control groups. Due to these characteristics, all selected studies had a high risk of bias, as assessed by the NOS scale (Table S2). Five studies were conducted in East Asia, while the other two studies were conducted in Germany and Turkey.

A total of 557 patients underwent Enterprise stent implantation for 588 ICAS lesions. The average age ranged from 56.8 to 64.0, and the pretreatment mean stenosis ranged from 65.4% to 92.0%. All studies reported some within 30 days after Enterprise implantation. Five studies, including 343 lesions, reported angiographic follow-up examinations, with the mean time ranging from 6 to 22 months. Five studies, including 370 lesions, reported results from clinical follow-up examinations, with the mean time ranging from 6.2 to 25.6 months.

#### 3.2.3. Immediate Angiographic and 30-Day Outcomes

**Figure 2:**
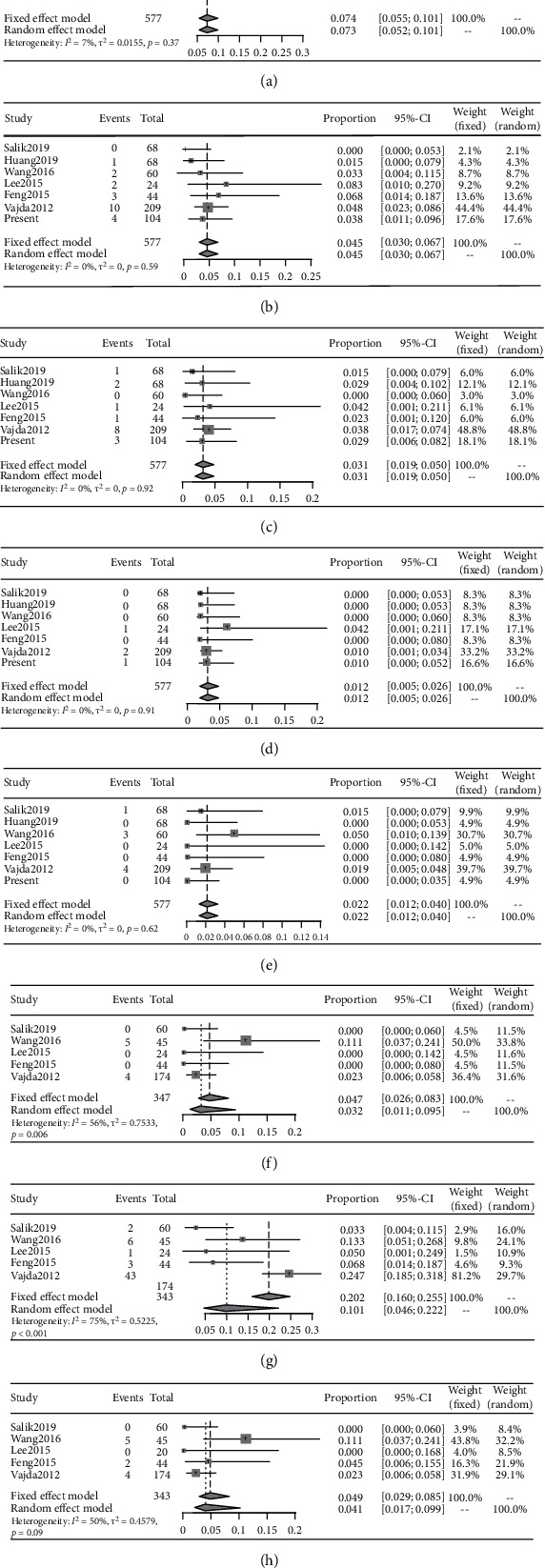
Pooled analysis outcome. (a) Any stroke or death within 30 days. (b) Ischemic stroke within 30 days. (c) Hemorrhage stroke within 30 days. (d) Mortality within 30 days. (e) Intraprocedure complication. (f) Ischemic stroke or TIA in the territory of the qualifying artery beyond 30 days. (g) In-stent restenosis during imaging follow-up. (h) Symptomatic in-stent restenosis during imaging follow-up.

The summary of adverse events after Enterprise implantation is shown in Table 3, and the forest diagram of the results is shown in Figure 2. The technical success rate ranged from 98.5% to 100%, with only one procedure that did not achieve technical success. Posttreatment stenosis was reported for 474 lesions in five studies, ranging from 12 ± 10% to 27.31 ± 8.89% (Table 2). Within 30 days following PTAS, the pooled incidence of adverse events was as follows: stroke or death, 7.4% (95% CI 5.5%–10.1%); hemorrhagic stroke, 3.1% (95% CI, 1.9%–5.0%); ischemic stroke, 4.5% (95% CI, 3.0%–6.73%); and mortality, 1.2% (95% CI, 0.5%–2.6%). The pooled incidence of intraprocedural complications was 2.2% (95% CI, 1.2%–4.0%), including vasospasm, hematoma in the groin, and asymptomatic dissection of the stented segment. These results were not heterogeneous (*I*^2^ = 0), and no apparent publication bias was observed in the funnel chart (Figure S2). We conducted a subgroup analysis of complications that occurred within 30 days by separating patients with lesions into AC and PC subgroups. The complication rate of patients with AC lesions was 6.7% (95% CI, 3.6%–12.3%), and for patients with PC lesions was 8.1% (95% CI, 4.9%–13.4%). There was no significant statistical difference between the two subgroups (Figure S3).

#### 3.2.4. Imaging and Clinical Follow-Up

Five studies, including 370 lesions, reported outcomes observed at clinical follow-up examinations. The pooled incidence of ischemic stroke or TIA in the territory of the qualifying artery beyond 30 days was 3.2% (95% CI, 1.1%–9.5%) (Figure 2). Since *I*^2^ = 56, we chose the analysis result obtained from the random-effects model. Through sensitivity analysis, we determined that the heterogeneity primarily resulted from the study by Wang et al. [15]. No deaths were reported during the follow-up examinations. Five studies, including 343 lesions, reported imaging follow-up results. The pooled incidence of ISR was 10.1% (95% CI, 4.6%–22.2%), and the pooled incidence of symptomatic ISR was 4.9% (95% CI, 2.9%–8.5%). The results of ISR were highly heterogeneous (*I*^2^ = 75). Using sensitivity analysis, we established that the heterogeneity principally resulted from the study by Vajda et al. [17]. We speculated that the heterogeneity might be caused by differences in the length of follow-up times. Based on the asymmetry of the funnel chart, we believe that the three results described above presented some degree of publication bias (Figure S2).

## 4. Discussion

We summarized our experience in a high-volume center and all published studies before May 2020 on the treatment of ICAS with the Enterprise stent and evaluated the safety and efficacy of the Enterprise stent. An analysis of 588 lesions in 557 patients revealed that the incidence of stroke or mortality within 30 days after the procedure was 7.4% (95% CI 5.5%–10.1%), and all but one procedure obtained technical success. In the SAMMPRIS and VISSIT trials, the incidence of adverse events within 30 days of PTAS with non-Enterprise stents was 14.7% and 24.1%, respectively, which are higher than the rates reported in this study. The long-term effect of treatment, as assessed by the incidence of ischemic stroke or TIA in the territory of the qualifying artery beyond 30 days, was 3.2% (95% CI, 1.1%–9.5%). Thus, our findings provided evidence to support the safety and effectiveness of Enterprise stent placement in the treatment of ICAS.

The endovascular procedure emerged as a novel ICAS treatment in the 1980s. Although technology and equipment have undergone constant innovation and improvement, the procedure has never become the predominant treatment for ICAS [26, 27]. Results of the WAISD and SAMMPRIS trials confirmed the safety of AMT [5]. However, for patients with high-grade stenosis, the rate of stroke recurrence after AMT was close to 20% per year [11]. For many patients with severe ICAS (stricture > 70%), PTAS treatment is still an important alternative treatment. Additionally, the results of the SAMMPRIS trial have been criticized by some experts due to limitations in stent selection, patient inclusion, and technical aspects of the procedures [28]. Therefore, exploring optimal treatments for ICAS is still a worthy endeavor.

After minimizing the limitations of the SAMMPRIS trial, an RCT in China, China Angioplasty and Stenting for Symptomatic Intracranial Severe Stenosis (CASSISS) [29], was initiated and is ongoing. Early reports from the CASSISS trial differed from the SAMMPRIS study results, including the observation that the incidence of 30-day adverse events in patients with high-grade ICAS treated by PTAS was only 4.3%. This result increased the confidence of practitioners to use PTAS for ICAS treatment in China. The extensive use of Wingspan stents in the SAMMPRIS trial also has been widely criticized [12]. Some experts believed that the rigidity and high radial force of the Wingspan stent were related to the high incidence of perioperative complications [30]. Recently, the Wingspan Stent System Post Market Surveillance Study (WEAVE) trial [31] and the Wingspan One-year Vascular Events and Neurologic Outcomes (WOVEN) trial [32] reported acceptable results. As postmarket surveillance studies, the WEAVE and WOVEN trials strictly enrolled patients treated on-label with the Wingspan stent and reported that 2.67% of patients had died or developed stroke within 72 hours, and 8.5% of patients had died or developed stroke at the one-year follow-up. These results suggest that when assessing the use of PTAS for ICAS treatment, the choice of an appropriate patient group is critical.

Following the failure of the SAMMPRIS and VISSIT trials, studies on the feasibility and effectiveness of using other alternative stents to treat symptomatic ICAS have been continuing [9, 13–20]. Among the many stent options, the self-expanding Enterprise stent has been used frequently for ICAS treatment, especially in our center. The Enterprise stent, which was specifically developed to treat wide-necked intracranial cerebral aneurysms, has a closed-cell design, special carrier system, and lower radial force compared with the Wingspan stent [15]. It has a diameter of 4.5 mm and has four lengths of 14, 22, 28, and 37 mm, so it is suitable for intracranial blood vessels with a diameter of 2.5-4.0 mm. The release rate of the Enterprise stent is less than 70%, and it is recyclable. More importantly, the Enterprise stent is exceptionally malleable, and its delivery catheter tip is soft and flexible, making it easier to reach the lesion area than more rigid stents [13]. Due to the wide application of the Enterprise stent to treat aneurysm embolization [33], it has been reported that it could reach areas inaccessible by other types of stents, such as the Neuroform EZ and Solitaire stents [34]. Interestingly, Vajda et al. proposed that the Enterprise stent could be delivered to any part of the circle of Willis with the aid of microcatheters [13].

Several previous studies reported low perioperative complication rates when using the Enterprise stent to treat ICAS that ranged from 1.47% to 12.50% [9, 13–17]. The results of this study agree with previous reports, as only 7.4% (95% CI 5.5%–10.1%) of patients experienced stroke or death within 30 days after Enterprise stent implantation. More adverse events were caused by ischemic events, 4.5% (95% CI 3.0%–6.7%), and although the incidence of hemorrhagic events was slightly lower, 3.1% (95% CI 1.9%–5.0%), death was always related to hemorrhagic stroke. Additionally, we conducted a subgroup analysis based on the AC/PC lesion location, and the complication rate was not significantly different between the two subgroups.

Several studies reported additional complications related to the procedure, such as vasospasm and stent migration [9, 13]. The periprocedural complication rates of the current study were undoubtedly better than the stent group of the SAMMPRIS trial but slightly higher than the WEAVE trial, the early results of the CASSISS trial, and the AMT group of the SAMMPRIS trial. These results could be related to patient selection, operator experience, and characteristics of individual PTAS. As the complication rate within 30 days after PTAS in the VISSIT trial was as high as 24.1%, the trial was terminated early. It is generally considered that the balloon-expandable stent is more rigid and less flexible than self-expanding stents and may be difficult to navigate along curved blood vessels [24]. Some experts believed that PTAS complications were related to the morphological classification of lesions, as proposed by Mori et al. [9, 14, 35], and the complication rates of type B and type C lesions were higher. Recently, a multicenter, single-arm study involving 159 patients explored the application of balloon-expandable stents in the treatment of ICAS. The complication rate at 72 hours after surgery was 0%, but the study included more Mori A (33.3%) and Mori B (52.2%) lesions, so the therapeutic effect of balloon-expandable stents for Mori C lesions is still worth exploring [36].

There are relatively few studies on other types of stents, so it is challenging to draw broad conclusions [18, 19]. A single-center study involving 76 patients explored the effect of a new generation of closed-cell self-expandable stents, the Acclino® flex stent, in the treatment of ICAS. The incidence of stroke or death within 30 days after PTAS was 6.5%, which is similar to the results of this study [19]. Also, another study reported that using the Neuroform EZ stent to treat ICAS has the possibility of reducing complication risks. That study included 71 consecutive patients, and no stroke or death was observed within 30 days after surgery. The open-cell design of the Neuroform EZ stent was thought to be associated with this result [18]. However, the exact risk factors for these perioperative complications after PTAS with the Enterprise stent are still uncertain, and further research is needed [37]. In particular, for high-grade stenosis, the low complication rate in our study supported the safety of Enterprise stent implantation in ICAS treatment.

For the follow-up results of the systematic review, the incidence of stroke, TIA, or death over 30 days after PTAS was 3.2% (95% CI, 1.1%–9.5%). This result was better than the AMT (6.4%) and stent groups (5.3%) in the SAMMPRIS trial and lower than the AMT (5.7%) and stent groups (12.1%) in the VISSIT trial. These results indicated that the long-term stroke prevention of the Enterprise stent was relatively good. Long-term complications are often associated with ISR, and the high incidence of ISR after stent implantation for ICAS has long been a major disadvantage of placing stents [38]. ISR is common in the first year after PTAS and an important cause of nonsurgical ischemic events after stent placement [10]. Importantly, we found that the ISR rate during the follow-up period after Enterprise stent implantation was 10.1% (95% CI 4.6%–22.2%), and the incidence of symptomatic ISR was 4.9% (95% CI 2.9%–8.5%). These results are in line with results from a previous meta-analysis of PTAS for ICAS treatment [39]. The Wingspan stent had an ISR rate between 6% and 42.8% [9], and the ISR rate was as high as 26.5% in the VISSIT trial using the balloon-expandable stent. The ISR rate in the study by Vajda et al. was 24.71%, which potentially could be related to the patient inclusion criteria and follow-up times, as numerous patients with a stenosis rate of 50%-70% were included and followed for a long time until ISR was detected [17].

Recently, a meta-analysis demonstrated that drug-eluting stents performed better in preventing ISR, with an ISR incidence of only 4.1% and an asymptomatic ISR incidence of 3.0% [20]. However, the risk of low-grade chronic inflammation leading to late stent thrombosis limits the application of drug-eluting stents. Moreover, drug-eluting stents are quite stiff, making it challenging to reach complex stenosis lesions, causing drug-eluting stents to be inferior to the Enterprise stent in terms of operability [20].

Several relevant studies have indicated that ISR may be related to lesion location, preoperative stenosis, use of a balloon, and the application of antiplatelet drugs [38]. However, few studies have focused on the mechanisms underlying ISR. A few studies have reported that the low radial force of Enterprise stents might play a role in reducing the ISR rate because it is related to intimal hyperplasia [33]. Currently, treatment for ISR includes medical therapy, bypass surgery, and endovascular recanalization, but most of these treatments have unsatisfactory outcomes [38]. In our center, the use of antiplatelet agents was strictly regulated based on the WASID trial to better prevent the emergence of ISR.

## 5. Limitations

Limitations associated with a retrospective single-arm study were unavoidable in the institutional series. Despite the authenticity of medical records, recall bias and selection bias were common, and the lack of a control group limited the scope of the results. For the systematic review, all included studies were retrospective single-center studies and lacked appropriate control groups for comparison with other treatments. The follow-up times and imaging methods for each study were highly variable, which may have confounded the clinical and angiographic results. The small number of available studies limited the effectiveness of evaluating publication bias. Also, this study only included relevant studies that took place in the last ten years. Over time, stenting technologies have continuously improved. The complication rate has decreased, which might be partially responsible for the heterogeneity in the results when studies published across a long-time span were compared. According to the Grading of Recommendations, Assessment, Development, and Evaluation framework, we found that the heterogeneity and methodological limitations of the included studies negatively impacted data in this study [40–42]. Nevertheless, with 557 patients and 588 lesions, this systematic review was the most extensive study to date that investigated the use of the Enterprise stent for ICAS. This study provided useful data to evaluate the effects of the Enterprise stent in the treatment of ICAS.

## 6. Conclusion

The use of the Enterprise stent for intracranial angioplasty may be safe and effective in the treatment of ICAS. When used appropriately, the vast majority of ICAS patients receiving Enterprise stent implantation obtained good outcomes and excellent neurological performance during the follow-up period. However, considering the limitations associated with the level of evidence in this study, additional RCTs are needed to further verify the effects of Enterprise stents for ICAS. Furthermore, the results of this study might provide assistance in the selection of stents for endovascular treatment of symptomatic severe ICAS.

## Figures and Tables

**Table 1:** The patient's demographic data, clinical and angiographic outcome.

Characteristic	Value
Age, years (mean ± SD)	58.61 ± 9.32
Sex, male/female (*n*)	60/44
Qualifying event (*n* (%))	
Transient ischemic attack	21 (20.19)
Cerebral infarction	83 (79.81)
Comorbidities (*n* (%))	
Hypertension	80 (76.92)
Diabetes	30 (28.85)
Coronary artery disease	18 (17.31)
Pretreated modified Rankin scale score (*n* (%))	
0	17 (16.35)
1	69 (66.35)
2	16 (15.38)
3	1 (0.96)
Location (*n* (%))	
Intracranial segment of internal carotid artery	13 (12.38)
Middle cerebral artery	34 (32.38)
Basilar artery	43 (40.95)
Intracranial segment of the vertebral artery	15 (14.28)
Lesion morphology (*n* (%))	
A	14 (13.33)
B	40 (38.10)
C	51 (48.57)
Complications (*n* (%))	
Any stroke or death within 30 d	7 (6.73)
Nonfatal ischemic stroke within 30 d	4 (3.85)
Nonfatal hemorrhage stroke within 30 d	2 (1.92)
Death within 30 d	1 (0.96)
Angiographic outcome	
Pretreated stenosis degree (%) (mean ± SD)	87.13 ± 7.80
Posttreated stenosis degree (%) (mean ± SD)	27.31 ± 8.89
Length of stenosis (mean ± SD)	10.68 ± 4.99
Enterprise stent length (mm) (mean ± SD)	25.12 ± 5.03
Balloon catheter length (mm) (mean ± SD)	13.22 ± 3.64

**Table 2:** Baseline characteristics of the patients and the lesions.

Study name	Period	Location	Design	No. of patients/lesions	Male/female ratio	Age (yr) (mean ± SD)	Stroke/TIA ratio	Lesion site AC/PC	Prestent stenosis rate (%) (mean ± SD)	Poststent stenosis rate (%) (mean ± SD)	Mean clinical or angiographic follow-up(months)
Vajda 2012	2007-2011	Germany	R	189/209	132/57	64	100/89	89/120	65.4 ± 0.8	25.1 ± 1.0	6.9
Feng 2015	2009-2013	China	R	44/44	32/12	60.45 ± 9.07	26/18	25/19	79.32 ± 8.18	NA	25.6
Lee 2016	2013-2014	South Korea	R	24/30	20/4	61.8 ± 10.3	NA	20/10	81 ± 11.3	18 ± 6.8	15.8
Wang 2016	2012-2014	China	R	60/62	42/18	56.8 ± 8.0	22/38	14/48	NA	22.8 ± 4.8	6.3
Huang 2019	2014-2018	China	R	68/70	46/22	59.43 ± 9.74	65/3	54/16	NA	NA	NA
Salik 2019	2012-2017	Turkey	R	68/68	56/12	62 ± 7	61/7	29/39	92 ± 6	12 ± 10	22
Present	2017-2020	China	R	104/105	60/44	58.61 ± 9.32	83/21	47/58	87.13 ± 7.80	27.31 ± 8.89	NA

yr: year; SD: standard deviation; R: retrospective study; NA: not available; TIA: transient ischemic attack; AC: anterior circulation; PC: posterior circulation.

**Table 3:** Summary of adverse events after Enterprise implantation.

Study	Intraprocedural complications	Any stroke or death within 30 days				Ischemic stroke or TIA in the territory of the qualifying artery beyond 30 days	Mortality beyond 30 days	ISR	
		Stoke		Nonfatal stroke	Death			Asymptomatic	Symptomatic
		Hemorrhagic	Ischemic						
Vajda 2012	4/209	8/189	10/189	16/189	2/189	4	0/174	39/174	4/44
Feng 2015	0/44	1/44	3/44	4/44	0/44	0	0/44	1/44	2/44
Lee 2016	0/30	1/24	2/24	2/24	1/24	0	0/24	1/20	0/20
Wang 2016	3/62	0/60	2/60	2/60	0/60	5	0/60	1/45	5/45
Huang 2019	0/70	2/68	1/68	3/68	0/68	NA	NA	NA	NA
Salik 2019	1/68	0/68	0/68	1/68	0/68	0	0/68	2/60	0/60
Present	0/105	3/104	4/104	6/104	1/104	NA	NA	NA	NA

NA: not available.

## Data Availability

The statistical results of our single center, as well as the data of the system review and the results of the pooled analysis, can be obtained in the manuscript and supplementary materials.

## Supplementary Materials

Supplementary Materials Table S1: database search strategy. Table S2: a modified version of the Newcastle-Ottawa Quality Assessment Scale. Figure S1: screening flowchart of the institutional series. Figure S2: funnel chart for detecting publication bias. Figure S3: subgroup analysis based on the anterior and posterior circulation of cerebral artery.
