# Supplementary material for: Intracranial Angioplasty with Enterprise Stent for Intracranial Atherosclerotic Stenosis: A Single-Center Experience and a Systematic Review
Source: Biomed Res Int. 2021 Apr 17;2021:6645500. doi: 10.1155/2021/6645500 (PMC8075681; doi:10.1155/2021/6645500)
Supplement: Supplementary Materials — Table S1: database search strategy. Table S2: a modified version of the Newcastle-Ottawa Quality Assessment Scale. Figure S1: screening flowchart of the institutional series. Figure S2: funnel chart for detecting publication bias. Figure S3: subgroup analysis based on the anterior and posterior circulation of cerebral artery. [file 6645500.f1.doc]

**Table S1. Search syntax**

| PubMed Search Accessed on May 1, 2020  (80 Articles) | EMBASE Search Accessed on May 1, 2020 (182 Articles) | Web of Science Search Accessed on May 1, 2020 (79 Articles) |
| --- | --- | --- |
| #1 intracranial arteriosclerosis [Title/Abstract]  #2 intracranial arteriosclerosis [Mesh]  #3 #1 OR #2  #4 cerebral arterial diseases [Title/Abstract]  #5 cerebral artery [Title/Abstract]  #6 cerebral arterial diseases [Mesh]  #7 cerebral arteries [Mesh]  #8 internal carotid artery [Title/Abstract]  #9 carotid artery, internal [Mesh]  #10 basilar artery [Title/Abstract]  #11 basilar artery [Mesh]  #12 vertebral artery [Title/Abstract]  #13 vertebral artery [Mesh]  #14 middle cerebral artery [Mesh]  #15 middle cerebral artery [Title/Abstract]  #16 #4 OR #5 OR #6 OR #7 OR #8 OR #9 OR #10 OR #11 OR #12 OR #13 OR #14 OR #15  #17 #3 OR #16  #18 stents [Mesh]  #19 stent [Title/Abstract]  #20 #18 OR #19  #21 Enterprise [Title/Abstract]  #22 #20 AND #21  #23 #17 AND #22 | #1 'intracranial arteriosclerosis’: ab,ti  #2 ‘intracranial AND arteriosclerosis’: ab,ti  #3 #1 OR #2  #4 'cerebral arterial diseases’: ab,ti  #5 'cerebral artery’: ab,ti  #6 'cerebral arteries':ab,ti  #7 'internal carotid artery’: ab,ti  #8 'carotid artery, internal’: ab,ti  #9 'basilar artery’: ab,ti  #10 'vertebral artery’: ab,ti  #11 'middle cerebral artery’: ab,ti  #12#4 OR #5 OR #6 OR #7 OR #8 OR #9 OR #10 OR #11  #13#3 OR #12  #14 ‘stents’: ab,ti  #15 ‘stent’: ab,ti  #16 #14 OR #15  #17 ‘enterprise’: ab,ti  #18 #16 AND #17  #19 #14 AND #18 | #1 TS= (intracranial arteriosclerosis OR intracranial arteriosclerosis stenosis OR intracranial stenosis OR cerebral arterial diseases OR cerebral artery OR cerebral arteries OR internal carotid artery OR carotid artery, internal OR basilar artery OR vertebral artery OR middle cerebral artery)  #2 TS= (stent OR stents OR stent*)  #3 TS= Enterprise  #4 #2 AND #3  #5 #1 AND #4 |

**Table S2. The quality measure of included studies by the Modified Newcastle-Ottawa Quality Assessment Scale: retrospective design**

|  | **Selection** | | | |  | **Outcome** | |  |
| --- | --- | --- | --- | --- | --- | --- | --- | --- |
| **Study Name** | **1** | **2** | **3** | **4** | **Comparability** | **1** | **2** | **Total** |
| Vajda 2012 | * | * | c | b | * | * | b | 4 |
| Feng 2015 | * | * | c | b |  | * | * | 4 |
| Lee 2016 | * | * | c | b |  | * | * | 4 |
| Wang 2016 | * | * | c | b | * | * | b | 4 |
| Huang 2019 | * | * | c | b | * | * | b | 4 |
| Salik 2019 | * | * | c | b |  | * | * | 4 |
| Present | * | * | c | b |  | * | b | 3 |

**Selection**

1) Is the case definition adequate?

a) Yes, with independent validation*

b) Yes, eg, record linkage or based on self-reports

c) No description

2) Representativeness of the cases

a) Consecutive or obviously representative series of cases*

b) Potential for selection biases or not stated

3) Selection of controls

a) Community controls*

b) Hospital controls

c) No description

4) Definition of controls

a) No history of disease (end point) *

b) No description of source

**Comparability**

1) Comparability of cases and controls based on the design or analysis

a) Study controls for _____ (select the most important factor) *

b) Study controls for any additional factor (these criteria could be modified to indicate a specific control for a second important factor) *

**Exposure**

1) Assessments of exposure

a) Secure record (eg, surgical records) *

b) Structured interview where blind to case/control status*

c) Interview not blinded to case/control status

d) Written self-report or medical record only

e) No description

2) Sufficient follow-up time

a) Yes*

b) No


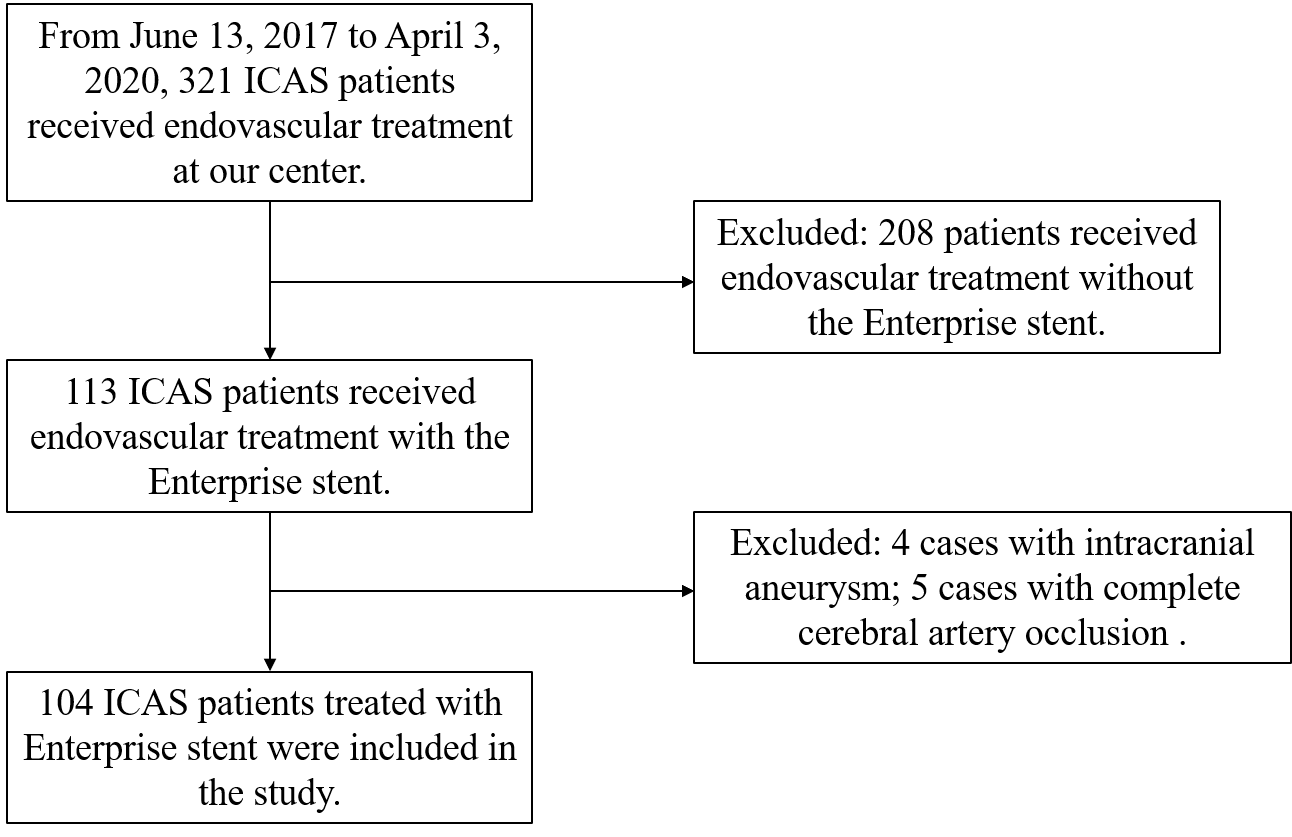


**Figure S1:** screening flowchart of the institutional series.


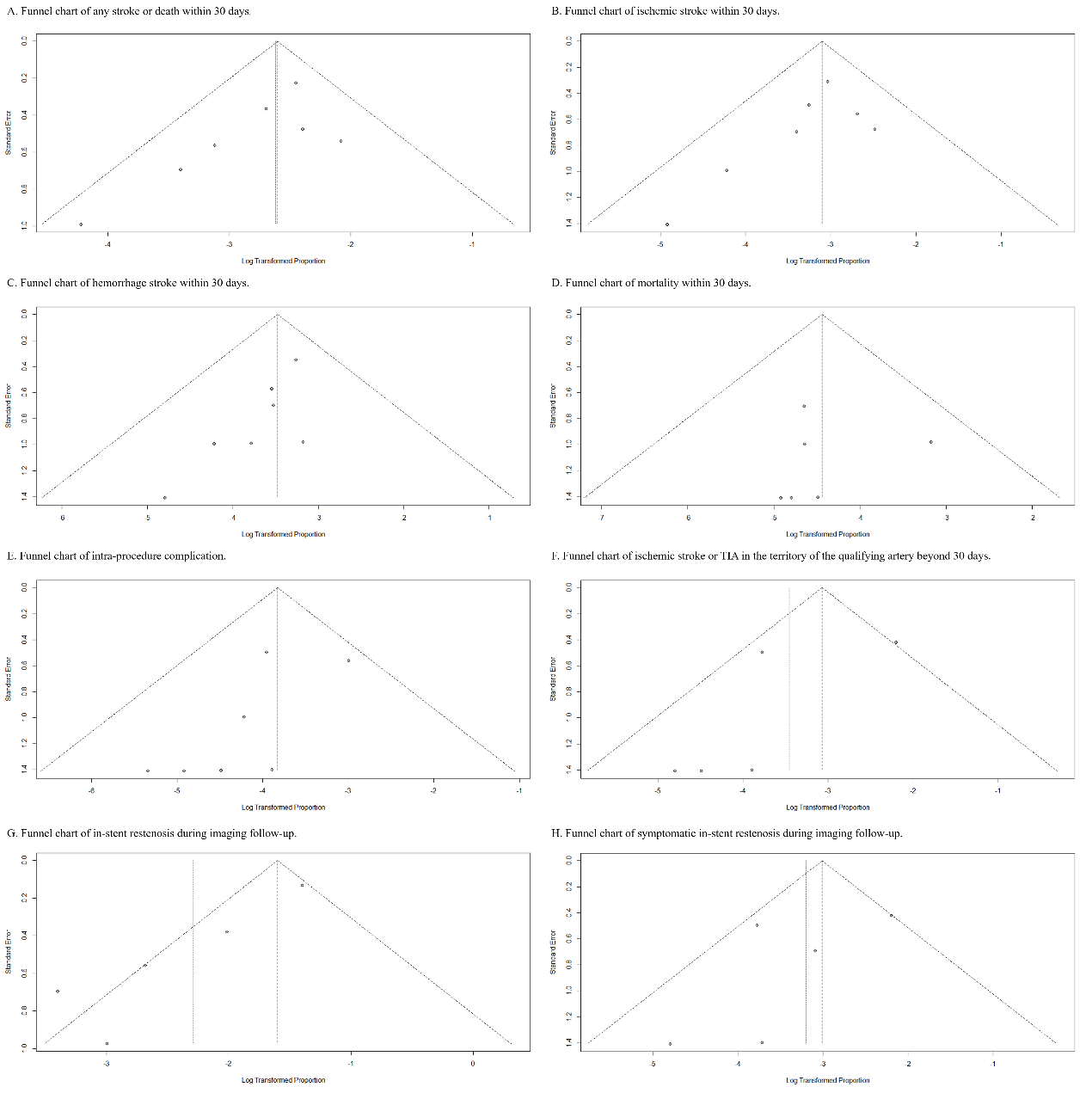


**Figure S2:** (A) Funnel chart of any stroke or death within 30 days. (B) Funnel chart of ischemic stroke within 30 days. (C) Funnel chart of hemorrhage stroke within 30 days. (D) Funnel chart of mortality within 30 days. (E) Funnel chart of intra-procedure complication. (F) Funnel chart of ischemic stroke or TIA in the territory of the qualifying artery beyond 30 days. (G) Funnel chart of in-stent restenosis during imaging follow-up. (H) Funnel chart of symptomatic in-stent restenosis during imaging follow-up.


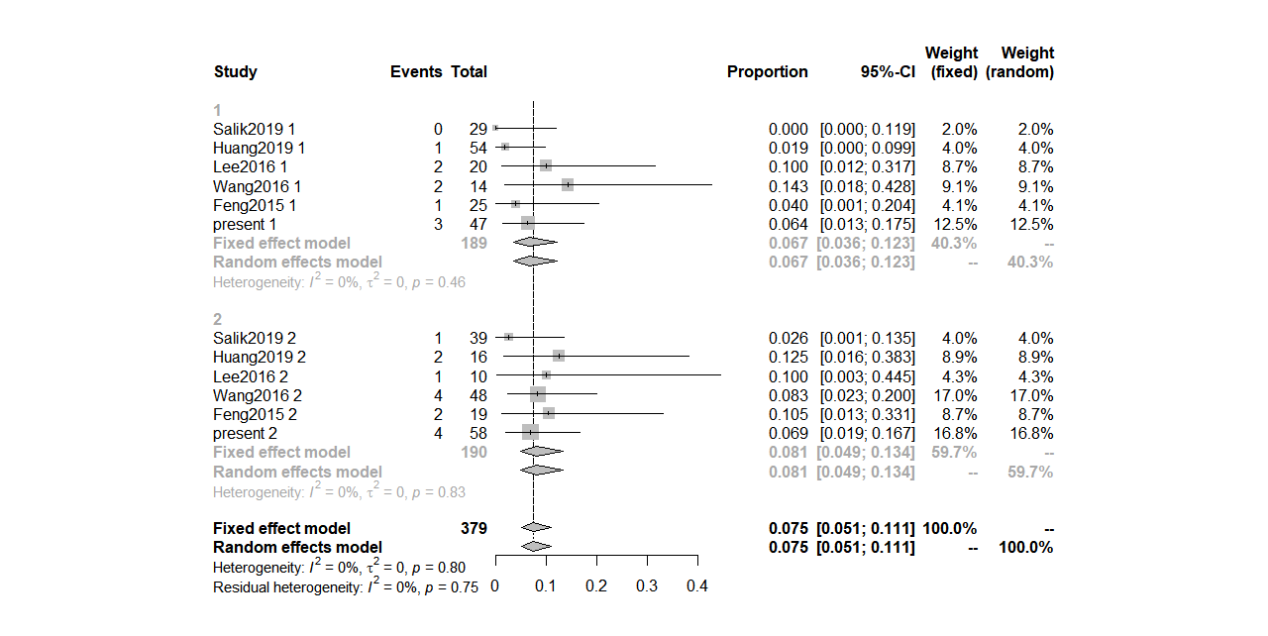


**Figure S3:** subgroup analysis based on anterior and posterior circulation of cerebral artery.
